# Biophysical Psychiatry—How Computational Neuroscience Can Help to Understand the Complex Mechanisms of Mental Disorders

**DOI:** 10.3389/fpsyt.2019.00534

**Published:** 2019-08-06

**Authors:** Tuomo Mäki-Marttunen, Tobias Kaufmann, Torbjørn Elvsåshagen, Anna Devor, Srdjan Djurovic, Lars T. Westlye, Marja-Leena Linne, Marcella Rietschel, Dirk Schubert, Stefan Borgwardt, Magdalena Efrim-Budisteanu, Francesco Bettella, Geir Halnes, Espen Hagen, Solveig Næss, Torbjørn V. Ness, Torgeir Moberget, Christoph Metzner, Andrew G. Edwards, Marianne Fyhn, Anders M. Dale, Gaute T. Einevoll, Ole A. Andreassen

**Affiliations:** ^1^Department of Computational Physiology, Simula Research Laboratory, Oslo, Norway; ^2^NORMENT, Division of Mental Health and Addiction, Oslo University Hospital & Institute of Clinical Medicine, University of Oslo, Oslo, Norway; ^3^Department of Neurology, Oslo University Hospital, Oslo, Norway; ^4^Department of Neurosciences, University of California, San Diego, La Jolla, CA, United States; ^5^Department of Radiology, University of California, San Diego, La Jolla, CA, United States; ^6^Martinos Center for Biomedical Imaging, Massachusetts General Hospital, Harvard Medical School, Charlestown, MA, United States; ^7^Department of Medical Genetics, Oslo University Hospital, Oslo, Norway; ^8^NORMENT, Department of Clinical Science, University of Bergen, Bergen, Norway; ^9^Department of Psychology, University of Oslo, Oslo, Norway; ^10^Faculty of Medicine and Health Technology, Tampere University, Tampere, Finland; ^11^Department of Genetic Epidemiology in Psychiatry, Central Institute of Mental Health, Medical Faculty Mannheim, University of Heidelberg, Mannheim, Germany; ^12^Cognitive Neuroscience Department, Donders Institute for Brain, Cognition and Behaviour, Radboud University Medical Centre, Nijmegen, Netherlands; ^13^Department of Psychiatry (UPK), University of Basel, Basel, Switzerland; ^14^Prof. Dr. Alex. Obregia Clinical Hospital of Psychiatry, Bucharest, Romania; ^15^Victor Babes National Institute of Pathology, Bucharest, Romania; ^16^Faculty of Medicine, Titu Maiorescu University, Bucharest, Romania; ^17^Faculty of Science and Technology, Norwegian University of Life Sciences, Ås, Norway; ^18^Department of Physics, University of Oslo, Oslo, Norway; ^19^Department of Informatics, University of Oslo, Oslo, Norway; ^20^Centre for Computer Science and Informatics Research, University of Hertfordshire, Hatfield, United Kingdom; ^21^Institute of Software Engineering and Theoretical Computer Science, Technische Universität zu Berlin, Berlin, Germany; ^22^Department of Biosciences, University of Oslo, Oslo, Norway

**Keywords:** genome-wide association study, computational modelling, ion channels, schizophrenia, psychotic disorders

## Abstract

The brain is the most complex of human organs, and the pathophysiology underlying abnormal brain function in psychiatric disorders is largely unknown. Despite the rapid development of diagnostic tools and treatments in most areas of medicine, our understanding of mental disorders and their treatment has made limited progress during the last decades. While recent advances in genetics and neuroscience have a large potential, the complexity and multidimensionality of the brain processes hinder the discovery of disease mechanisms that would link genetic findings to clinical symptoms and behavior. This applies also to schizophrenia, for which genome-wide association studies have identified a large number of genetic risk loci, spanning hundreds of genes with diverse functionalities. Importantly, the multitude of the associated variants and their prevalence in the healthy population limit the potential of a reductionist functional genetics approach as a stand-alone solution to discover the disease pathology. In this review, we outline the key concepts of a “biophysical psychiatry,” an approach that employs large-scale mechanistic, biophysics-founded computational modelling to increase transdisciplinary understanding of the pathophysiology and strive toward robust predictions. We discuss recent scientific advances that allow a synthesis of previously disparate fields of psychiatry, neurophysiology, functional genomics, and computational modelling to tackle open questions regarding the pathophysiology of heritable mental disorders. We argue that the complexity of the increasing amount of genetic data exceeds the capabilities of classical experimental assays and requires computational approaches. Biophysical psychiatry, based on modelling diseased brain networks using existing and future knowledge of basic genetic, biochemical, and functional properties on a single neuron to a microcircuit level, may allow a leap forward in deriving interpretable biomarkers and move the field toward novel treatment options.

## Background: Disparate Progresses in Different Fields of Medicine

Mental illnesses place a large emotional, health, and financial burden on patients, their families, and the society (1). Mental disorders account for about one third of all years lived with disability worldwide (2) with rising prevalence (3), and there is overwhelming evidence of a large mortality gap between individuals with mental illness and the general population (4, 5). At the same time, there have been limited improvements in treatment of affected individuals over the past decades, and there are no diagnostic or prognostic biomarkers for these disorders. Crucially, most mental illnesses are not caused by a single condition but arise from a complex interplay between several internal and environmental factors, and this makes drug development challenging (6). As a result, most major pharmaceutical companies have left this research field due to lack of future potential in standard research approaches (7). These shortcomings have been highlighted by the European Union and the World Health Organization, which have called for additional research to address this problem (www.who.int/mental_health/mhgap/en/).

In other fields of biomedicine, significant progress has been made during the last decades. There has been a continuous reduction in the mortality rate of heart disease since the 1970s, and the mortality rate for several types of cancer has also started to decline (8, 9). A key development in recent years was the completion of the Human Genome Project in 2003 (10), which marked a beginning of a new era (11, 12). Important subfields of biology, such as structural and functional genomics, systems biology, and statistical genetics, have emerged to figure out the implications of the genetic content on living cells and how the genes play together to cause cellular and tissue-level phenotypes as well as human diseases (11).

In parallel, in the wake of efficient computer technology and advanced software, the use of computational models for describing neuronal function has become an important tool for understanding the behavior of single neurons and neuronal networks (cf. 13, 14). Detailed and compartment-specific models of neurons describe the transmembrane currents of different ion-channel families and their voltage-dependent gating, and they are typically based on real (three-dimensionally reconstructed) neuron morphologies, although reduced compartmental models can also be used for computational efficiency or generalizability (15). Moreover, the modelled neurons can be coupled with each other using descriptions of synaptic currents to create biophysical models of large-scale neuronal circuits (16). To standardize the methodology in computational neuroscience and to facilitate reproduction of simulation results, efficient simulation software has been developed, such as NEURON, which is a widely used simulator of single- and multi-compartment neuron models and model networks that flexibly allows the user to define the ion channels and their distribution along the neurites (17). These advances allow analyses of how alterations in a specific ion-channel family can affect the functions of the neural circuit at local and global scales and can thus foster many types of computational studies of heritable (or by other means of genetic origin, i.e., through *de novo* mutations) mental disorders (18–20).

At present, the role of specific signaling molecules and ion channels in mental disorders is poorly understood, and most of the specific hypotheses regarding, for example, molecular genetic factors and psychiatric disease will have to be considered tentative and preliminary. However, we are now getting to the situation where the computational modelling approaches will be sufficiently developed so that candidate hypotheses can be tested against experiments. Specifically, simulations of brain networks based on biophysically detailed neuron models are now becoming feasible, and molecular effects on the behavior of neurons and networks, and eventually also systems, can be systematically explored with mathematical modelling (14). This could be compared with the progress of weather forecasting technology. The knowledge about meteorological factors affecting the weather was defined already in the 1920s, but the computational power did not become available until the 1960s and 1970s, when weather forecasts, supported by improved parametrization of the underlying physical processes, started to become accurate (21). Today, we can accurately predict the weather for 1 week using advanced computational models that integrate meteorological and topographical data. While we do not yet have a single strong hypothesis of the underlying “microscopic principles” for mental disorders, the possibility to test, falsify, and refine present and future candidate hypotheses for these principles by comparing simulation results with physiological experiments could be a game-changer. Thus, building on genetic data, neuroscience research, biophysical insights, and large-scale computation, we might hope for a breakthrough in the understanding of the molecular and cellular mechanism behind mental disorders.

In the following, we review the relevant advances made in functional genomics, statistical genetics, and cellular neuroscience, and we suggest how these new data can be used in a computational neuroscience approach to understand mental disorders. Basing on the large increase in computation power and availability of quantitative data in molecular and cellular biology over the past years, we predict a wide range of possibilities for this “biophysical psychiatry” approach. Our main focus is on distorted functions of ion channels and disease phenotypes emerging from these alterations. Dysfunctional neuronal excitability represents the branch of mental disorders that is most ready for a prominent computational analysis, but we extend our discussion on other aspects of heritable mental diseases, which will be possible to simulate in the near future. We also discuss the most significant gaps of knowledge and obstacles for this approach.

## The Power and Shortcomings of Genome-Wide Association Studies

Genome sequencing data are an ideal source of information for large-population studies for at least three reasons. First, the data obtained are *independent of the state* (e.g., fatigue, mood, and phase in daily routines) of the subject and, apart from cancerous tissue, *generalizable* to any cell type in the human body [although data challenging this view do exist (22, 23)]. Second, the data obtained are *absolute* (no scaling or other preprocessing is needed), although several sources of error exist [see, e.g., Ref. 24)]. Third, it is relatively *inexpensive* to both take the sample and perform the genome-wide screening, at least if we concentrate on the commonly used single-nucleotide polymorphism (SNP) arrays (25). Consequently, genome-wide association study (GWAS) sample sizes have grown to tens of thousands of subjects, increasing the statistical accuracy of the obtained results.

These advantages have made possible the application of GWAS to a lot of heritable traits and diseases, including mental disorders. Crucially, mental disorders are polygenic phenotypes, that is, no single gene determines the disorder outcome, but the risk of the disorder may depend on as much as hundreds of genetic loci. For example, for schizophrenia, an ever-increasing number of genetic loci are identified: 108 genetic loci were identified using a sample of 37,000 affected individuals and 113,000 controls (26), and the number of identified risk loci was yet larger (145) in the latest GWAS (27). Importantly, the new GWAS results typically replicate the majority of the previous GWAS findings, which is often not the case with the hypothesis-driven candidate gene methods that used to be the standard in the field (28). The large number of identified risk variants associated with mental disorders and their frequent prevalence in healthy population (most of the identified gene variants are common) place a challenge on the functional genomics approach typically applied to variants of heritable diseases. The identified 145 schizophrenia loci typically contain many risk SNPs with variable degrees of linkage disequilibrium with each other. Application of novel biostatistics tools will likely capture more of the heritability of mental disorders and thus further increase these numbers (29–31). Another related challenge is the functional diversity of the identified loci. In schizophrenia, the implicated genes contribute to the immune system (32), neuronal electrogenesis (33, 34), synaptic function and neurotransmission (35), and redox homeostasis (36, 37).

Thus, a systematic functional genomics approach to polygenic mental disorders would have to 1) consider many cellular phenomena that a variant may affect, each of which should be particularly designed to quantify a phenotype in the underlying genetic pathway, 2) test many genetic loci to capture the effects of all risk variants in the considered gene, and 3) perform the experiments with large sample sizes to detect small effects, as expected from common variants. Overcoming these three challenges is beyond the capabilities of the scientific community of today. And if it becomes possible, we would still be left with the question on how the *interaction* between different variants is involved in inducing disease phenotypes. Although this certainly does not altogether disqualify reductionist methods driven by genomic data, it points us toward alternative approaches that allow making and testing hypotheses on the disease mechanisms of polygenic mental disorders, ideally in a less costly but as standardized and reproducible manner as the functional genomics approach. In the following sections, we will discuss how well-suited biophysically detailed computational modelling is for this purpose.

## The Computational Psychiatry Approach

Computational neuroscience builds upon describing of neurons, neuronal circuits, brain areas, or the whole brain by quantitative, computational models. On the single-neuron level, a computational model of a neuron is typically a set of equations that describes its properties based on a solid biophysical foundation—the current balance across the cell membrane and the conduction of ions across the media. Biophysically detailed whole-neuron modelling relies on two scientific breakthroughs in the last century [reviewed in Ref. (38)]. First, the work by Hodgkin and Huxley (39) to characterize and quantify the properties of action potential generation is still today a cornerstone of neuron modelling. Second, the work by Wilfrid Rall (40) to describe the signal propagation along neurites (the “cable theory”) forms a basis for multi-compartmental neuron modelling and the use of reconstructed neuron morphologies. The full potential of these two theoretical approaches has become (and is still becoming) possible through recent development of computer hardware and a base of knowledge about different ion channels and their functions. Indeed, Hodgkin and Huxley themselves could not reach their goal of characterizing the molecular basis of the action potential generation, but 20 years later, the discovery of ion channels established this basis [reviewed in Refs. (41, 42)].

During the 1980s and 1990s, a deeper characterization of the contributors to neuronal electrogenesis was obtained through the advances made in ion-channel blockers and electrophysiological and optical techniques, which allowed the construction of biophysically more detailed neuron models (43). Following the sequencing of the mammalian genomes, a focus is now on describing the ion-channel behavior in terms of their genetic composition. Online databases, such as Channelpedia (http://channelpedia.epfl.ch/) and ICGenealogy (https://icg.neurotheory.ox.ac.uk/) are examples of sources for such information (44, 45). Due to difficulties in both experimental design (46) and the model fitting (cf. 47, 48), this line of development is still in its infancy (45). Nevertheless, the existing biophysically detailed neuron models already classify different types of transmembrane currents based on their sensitivity to different blockers, and it is relatively well known which ion-channel subunits contribute to which of these currents (43). This lays a foundation for analyses of how different genes—and their different variants—affect the neuronal excitability on a cellular level.

### Capturing Effects of Genetic Alterations on Channel Properties in Biophysically Detailed Models

Data from functional genomics have attracted modellers of neurons and other excitable cells ever since they became available (49). Clancy and Rudy (50) modelled the effects of long-QT syndrome risk variants on cardiac ventricular action potentials basing on data from electrophysiological measurements performed on cells with wild-type or mutated channels. Splawski et al. (51) measured the effects of a Timothy syndrome mutation on the inactivation of the L-type Ca^2+^ channels and modelled their effects on cardiomyocyte action potentials. Spampanato et al. (52) predicted altered firing thresholds and increased firing rates for epilepsy-associated mutations based on data from electrophysiological measurements. These are a few of numerous examples, but a notable trend is that most of them concern cardiac cells and heart diseases rather than neurons and brain disorders. This may partly be due to the fact that cardiac disease constitutes the largest cause of death (thus representing an ultimately important topic) and partly because the cardiac cell models are in general genetically better characterized than the neuron models and the link between cellular and tissue-level pathologies is better understood (cf. 53). Among the brain disorders, epilepsy is likely to be the one that has been most studied using computational modelling, due to the multitude of scales at which the disease symptoms and phenotypes can be both observed and modelled (54, 55)—for a review of computational modelling strategies in this field, see Soltesz and Staley (56) and Wendling et al. (57).

In the brain, there are a vast number of different neuron types (let alone glial and endothelial cells), each of which expresses a different set of ion channels and other proteins that change with the developmental stage of the cell and its involvement in neuronal network dynamics (e.g., long-term plasticity) (58, 59). Consequently, each neuron type may contribute in a unique and dynamic way to brain disorder phenotypes. Such a complexity, added to the challenges caused by the branching neurite geometry (60), is a likely cause for a slower development of highly detailed single-neuron models than is the progress made in biophysical models of cardiac cells [see, e.g., Ref. (61)]. Nevertheless, certain neuronal cell types are already relatively well characterized in terms of biophysically detailed models, offering a platform for modelling functional genomics data. Two such neuron types are the Purkinje cells in the cerebellum (62, 63) (cf. 64) and the layer V pyramidal cells in the neocortex (65). Building upon a long line of Purkinje cell models [reviewed in Ref. (66)], a recent model (67) described the kinetics of 15 gene-based types of ion channels and their (manually fitted) distributions along the neuron morphology. The ion-channel descriptions in models of layer V pyramidal cells are not as well characterized down to genetic level, but the more recent models [following the principles in an early, biophysically detailed model by (65)] included nine to 13 different ion channels (68–70). In particular, these models included a simple description of the intracellular Ca^2+^ dynamics, differentiated between low- and high-voltage-activated Ca^2+^ currents, and described the kinetics of the Ca^2+^-activated K^+^ channels underlying the medium or slow afterhyperpolarization. These models can thus be used to analyze the contributions of both ion channel- and Ca^2+^ transporter-encoding genes to hyperpolarizing and depolarizing transmembrane currents—and thus to neuron firing.

Unlike simpler models such as integrate-and-fire models or the basic two-channel Hodgkin–Huxley model (39), the biophysically detailed models enable analysis of many aspects of neuron excitability and the contributions of different genes and gene variants to these properties. Masoli et al. (67) validated the model against data from genetic studies that knocked out certain ion channel-encoding genes and observed effects on spontaneous and stimulated firing behaviors, which argues for the usability of the model in predicting effects of mutations in the underlying genes.

### Modelling of Non-Ion Channel-Related Genetic Variants and Other Disease-Related Alterations

Rather than simple genetic channelopathies, heritable mental disorders are typically hypothesized to be complex diseases affected by genetic variants in a variety of gene ontologies as well as by environmental factors. To this end, many types of genetic perturbations, other than variants of neuronally expressed voltage-gated ion channel-encoding genes as outlined above, can be flexibly included in computational studies of heritable mental disorders. The premise is that neuronal signalling is the common pathway for all brain biology generating thoughts, emotions, and behavior, and therefore, dysfunctional or lacking neuron firing in one form or another is a common abnormality in all mental diseases. A typical solution for modelling mental diseases other than channelopathies is to use less biophysically detailed modelling approaches, where a disease-related condition observed at a cellular or network level (instead of at the level of proteins) is implemented, and the effects of the condition on the network dynamics or circuit functions are predicted. To name a few examples, in the modelling study of Vattikuti and Chow (71), the autism spectrum disorder-associated phenotypes of saccade hypometria and dysmetria were shown to result from a distorted excitation/inhibition balance, which is one of the leading hypotheses for network-level aberrations in autism spectrum disorder. In a similar fashion, NMDA receptor hypofunction—a widely hypothesized cellular mechanism in schizophrenia [reviewed in Ref. (72)]—was shown to lead to a schizophrenia-associated phenotype, namely, distortions in gamma band oscillations, in a series of modelling studies [reviewed in Ref. (73)]. Comparable computational network studies have used as a starting point a decreased intensity of GABAergic neurotransmission, a schizophrenia anomaly widely suggested by post-mortem studies (74, 75), and analyzed its effects on network dynamics in light of schizophrenia-associated phenotypes (76–79) or hypothesized modes of psychotic circuit activity (80). Similar strategies have been employed in examining the effects of hypodopaminergic modulation of prefrontal cortex, one of the more traditional hypotheses for schizophrenia mechanisms (81), on working memory capacity and precision (82). Typically, these studies employ simpler point-neuron models such as integrate-and-fire models, and the alteration of a model parameter used for representing the disease cannot be directly mapped down to gene level. However, new genetic data and understanding of genetic interactions could be used to revise these descriptions, which would lead to a better understanding of the heritable component of these disorders.

Another new promising subfield of computational psychiatry is the biochemically detailed modelling of brain disorders. For example, computational modelling has clarified the effects of Alzheimer’s disease-related genetic variants on β-amyloid plaque formation and tau-protein phosphorylation, which are among the main hypothesized causes for Alzheimer’s disease [see Ref. (83) for a review]. In Sasidharakurup et al. (84), intracellular signaling pathways in control vs. Parkinson’s disease cases were simulated, and the mechanisms leading to cell death were analyzed. Biochemically detailed modelling of immune system pathways may also constitute an important branch of future computational modelling work in the pathology of schizophrenia and autism, both of which have recently been associated with alterations in inflammatory pathways [reviewed in Refs. (85) and (86)]. A challenge in this type of models is the lack of cellular-level data with which to validate the model parameters describing the biochemical reactions as well as a lack of frameworks in which the model predictions in the cellular and subcellular regimes can be linked to system-level behavior. Nevertheless, such models may be a valuable aid in integrating the effects of environmental factors, including stress, with genetic factors to create a clearer picture of the pathogenesis of heritable mental disorders.

### Biophysical Psychiatry: Biophysically Detailed Modelling of Heritable Mental Disorder Mechanisms

**Figure 1:**
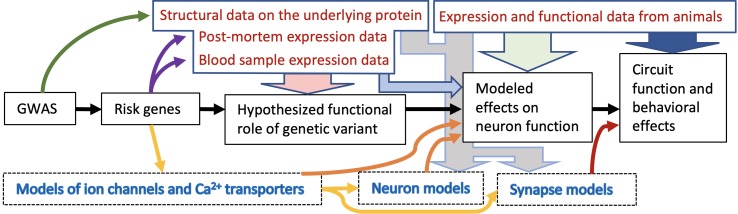
Schematic illustration of the biophysical psychiatry approach for studying heritable mental disorders. This approach typically consists of five main stages ranging from initial GWAS to prediction of the effects of genetic variants on circuit functions (black arrows). First, the results from GWASs are used to identify risk variants and risk genes. Data from patients and structural genomics can be used to constrain the range of possibilities how these variants affect the protein functions: Is there evidence of altered expression of these proteins in the patients and in what tissue (purple arrows)? Are the identified SNPs likely to affect the protein structure (dark green arrow)? This type of information is important for formulating an initial hypothesis on the functional role of the variant (light red arrow), and importantly, together with data from functional genomics, it can be used for computational models of neurons or ion channels as well as other proteins affecting the function of the neuronal circuit (yellow arrows). The effects of the risk gene variants on neuron function can be predicted with the models (orange arrows). When these effects are implemented in a network model that describes both neuronal activity and synaptic communication between the neurons, the impact of the variants on circuit and/or behavioral functions can be predicted (dark red arrow). In case the genetic background of the disease is not known or it is not possible to directly link the genetic variant effects to all desired aspects of the model behavior, cellular-level data from patients or animal model studies can be used as a shortcut and make the model express the dichotomy suggested by the experiments (light grey arrows). Lastly, data from animal studies can be used to validate the model: Is the studied neuron type really behind the modelled phenotype (light green arrow)? Are the genes in which the modelled variants are located expressed in the particular neuron type (light blue arrow)? Are there behavioral observations in animal model studies that support the conclusions (dark blue arrow)? These are but few questions to consider in a modelling approach bridging the gap between genes and behavior.

We propose to combine neuroscience modelling with psychiatric genomic data in an approach we have termed “biophysical psychiatry.” This is a particular type of neuroscience modelling that integrates data on risk genes and variants thereof to study the pathology of mental disorders. We propose that the development of biophysically detailed models is a suitable method for creating hypotheses on the mechanisms of heritable mental disorders. These can be tested and validated in experiments, both preclinically on a (translational) functional level and clinically, and then further refined. We illustrate the overall concept in **Figure 1**.

In our previous work, we applied a polygenic modelling framework similar to that of **Figure 1** to study the effects of small effect-size variants of schizophrenia-associated genes on layer V pyramidal cell excitability and integration of inputs (20, 87). Another study measured the effects of schizophrenia-associated *de novo* mutations in *CACNA1I* gene on the surface expression of the encoded protein and the corresponding Ca^2+^ current and simulated their effects on neuron firing in the thalamic reticular nucleus (88). In a modelling study of major depressive disorder, Ramirez-Mahaluf et al. (89) showed that a slowed-down reuptake of glutamate [an alteration supported by post-mortem experimental data from major depressive disorder patients (90)], caused hyperexcitability in the ventral anterior cingulate cortex and suggested an impairment of switching from emotional to cognitive processing. A study combining experimental data and computational modelling showed that a reduction of SK currents, which was experimentally shown to follow a loss-of-function mutation of *SCN1A* in an epilepsy mouse model, led to prolonged seizure-like bursts of reticular thalamic cells (91). Importantly, when analyzing the effects of the mutations on network dynamics, the biophysically detailed models, unlike integrate-and-fire models and rate-based models, enable the separation between voltage-gated ion channel-mediated and synaptic scaling-mediated effects. For example, we recently showed that neither synaptic scaling [such as that provided by homeostatic plasticity (92)] nor an artificial manipulation of the passive membrane properties can imitate the predicted effects of our variants of voltage-gated ion channel- and Ca^2+^ transporter-encoding genes on delta power (93). Although such results could not be obtained using simple integrate-and-fire models, these simpler models are nevertheless invaluable in analysis of large-scale network activity. Previously, innovative approaches combining the scalability of the simpler models and the biophysical detail of the more complex models have been applied to the forward modelling of local field potentials (94) and analysis of neuronal input–output relationships (95) and could be employed in studying the genetic effects as well.

### Top-Down and Bottom-Up Modelling Approaches

The approaches above can be considered *bottom-up* approaches as their foundation lies on models and data in the cellular and genetic levels, and they make predictions for higher-level phenotypes. Nevertheless, important insights can also be obtained from *top-down* approaches, where the models are fitted to higher-level data, such as electroencephalography (EEG) or functional magnetic resonance imaging (fMRI) data, and can make predictions for the cellular of sub-cellular level phenotypes. Typically, bottom-up models are founded in basic laws of physics, while top-down models are more phenomenological by nature. As an example of a top-down approach, in dynamic causal modelling (DCM), a neural mass model is constructed based on EEG or fMRI data and an underlying network model (96, 97). Given measurements of both mental disorder patients and healthy controls, this type of modelling can reveal important circuit pathologies underlying the mental disease. In one study based on DCM and EEG data from patients with psychosis and healthy controls performing an oddball task, a decrease in frontal inhibitory connections in patients with psychosis was predicted (98). Another DCM study predicted an impaired thalamocortical connection in schizophrenia basing on fMRI data collected from psychotic patients and healthy controls performing a verbal fluency task (99). However, the use of DCM for fMRI data has been questioned due to challenges in the underlying biophysical model and statistical inversion (100). Many other biophysically less detailed modelling approaches exist as well, as reviewed in Montague et al. (101) and Wang and Krystal (102). These approaches are especially useful in studying the pathology of non-heritable mental disorders, where the cause of the disorder may be dysfunction or dysconnectivity in a single brain region—however, the use of models that are more detailed, all the way to the genetic level, can be argued for when studying the cause of heritable mental disorders.

### Limitations of Biophysical Models of Psychiatric Disease—and Opportunities

There are several challenges in the biophysical psychiatry outlined above. First, the risk variants are typically identified by tag SNPs, which represent variations within a region of DNA instead of a single causative SNP (103). This may distort the analysis of the effects of the variant, as the causal SNP may reside within or outside the protein-coding region of a gene or may regulate many distinct genes. Second, as mentioned earlier, the computational models made for ionic currents are usually not unique to ion-channel subunits encoded by a single gene, and even if they were, there are many parallel genetic pathways that affect the channel kinetics and maximal conductance. While the ion-conducting pore in voltage-gated Ca^2+^ and Na^+^ channels is composed of a single α subunit with repeating motifs, the pore in the K^+^ channels is composed of four subunits that may (homomeric) or may not (heteromeric) be identical, and the composition typically impacts the conductance, voltage dependence, or kinetics of the channel (104). Furthermore, the expression of voltage-gated K^+^ as well as Ca^2+^ and Na^+^ channels on the membrane and their functional properties is affected by the auxiliary subunits (e.g., β subunits) (105). This presents a particular challenge for modelling of polygenic diseases, such as schizophrenia, where there are identified gene variants in various subunit-encoding genes even of the same ion-channel family (34). Third, the neuron models are typically built and validated using data from a specific brain region of a particular animal species (typically, rat or mouse), and thus, predictions made for human disease phenotypes based on such models unavoidably represent a generalization of a kind (106). Fourth, since the models are typically built using single-cell data only, their predictions are primarily applicable to cellular phenotypes only, and prediction of brain-level phenotypes requires careful adjustment of the network model according to the underlying brain microcircuits (107). In particular, the heterogeneity of macroscopic brain signals, such as EEG, in both spatial and temporal domains makes it difficult to link cellular phenotypes to clinical observations (108). This presents a challenge for psychotic disorders, where the symptoms and phenotypes are complex and the cellular and network mechanisms underlying them are largely unknown (109).

Nevertheless, there are ongoing large-scale projects and recent technological advances that aid in overcoming the abovementioned obstacles. First, ongoing international projects such as ENCODE as well as new data from deep sequencing (110) offer new detailed insights on the genetic risks of diseases. This can help in identifying risk-conferring SNPs and discovering the correlation structure between the SNPs, both locally and across the genome, and thus complements the GWAS data from large consortia. Second, quantitative data on ion-channel behavior and the contributions of different subunits and other proteins are rapidly growing, thanks to both online databases (such as Channelpedia and ICGenealogy) and new insights from molecular dynamics simulation approaches (111). Third, new large-scale projects such as the Allen Brain Atlas (https://www.brain-map.org/) and Human Protein Atlas (https://www.proteinatlas.org/) offer unique, standardized data sets on the expression of genes in both mice and men. In addition to brain area-wise information on gene expression and connectivity in the mouse brain, the Allen Brain Atlas includes a detailed data set on expression of tens of thousands of genes in different layers of the mouse primary visual cortex (http://casestudies.brain-map.org/celltax). This allows modellers of effects of particular gene variants on cortical phenotypes to check whether the underlying gene and related genes are expressed in the cell type they are interested in or not, which helps in formulating and refining the research hypotheses. Moreover, neuroinformatics databases such as ModelDB (https://senselab.med.yale.edu/modeldb/) help the modelers by offering curated neuron models from a variety of brain areas. Fourth, there are ongoing and recently completed large-scale projects that extend our knowledge on structure and function of brain circuits, in terms of both quantitative, computational models and qualitative or conceptual models. The NIH BRAIN initiative is a mega-scale program aiming at this goal (112). The Human Brain Project (https://www.humanbrainproject.eu/) has a comparable goal in developing an infrastructure that allows multi-scale modelling of large brain networks (113). Supporting this goal, the preceding EPFL-led project, the Blue Brain project (lending its name from the IBM’s Blue Gene supercomputer project) already offers large amounts of data that are directly usable in models (114). Improved quantification and understanding of the structure and dynamics of the neural circuits are essential for the accuracy of forward models of macroscopic brain signals and thus vital to the translation of findings from the models into clinical knowledge.

The projects named above support the development of new neuron models that are more accurate than before, in terms of both single-cell structure and dynamics (which ion channels are expressed and where in the neuron) and network interactions (how the neuron activity is affected by inputs from other cells). This is an important milestone on the way toward better predictive power for maps from gene to network level (107)—yet one has to keep in mind that detailed biophysics in one domain, such as voltage-gated ion channels, does not mean that the model is biophysically detailed in all aspects (cf. 19, 102). In particular, there is much to improve in how the actions of neuromodulators can be taken into account in the neuron models (115). Emphasis should also be placed on validation of the generated neuron models, which has recently been made easier by new, standardized tools (116).

In addition to these challenges, there is a lack of understanding of contributions of intracellular signalling mechanisms (117) as well as glial cell functions (118) and adult neurogenesis (119) to brain functions in health and disease. The schizophrenia-associated genes encode many types of phosphatases and kinases involved in neurotransmission and long-term plasticity (35)—variants of these genes can thus have crucial effects on neuronal circuit functions. Moreover, some of the schizophrenia-associated ion channel-encoding genes are also expressed in astrocytes (120). Biophysical models of glial functions, particularly models for Ca^2+^ excitability, exist, but the field is immature, and models are required to be better validated against electrophysiological and imaging data [for a review of models and their characteristics, see Ref. (121)]. The complexity of the intracellular signalling pathways also manifests in compensatory mechanisms that may counteract the effects of the modelled variants on both genetic [see, e.g., Ref. (122)] and cellular and network levels (123). Better integration across different scales and expanded quantitative descriptions of the biochemical machinery have been proposed as a path toward improved predictive power of the biophysically and biochemically detailed models (124, 125).

## Parallel Approaches in Computational Cardiac Science

As mentioned earlier, computational approaches have proved successful in reconstructing and explaining the mechanisms of several genetic conditions in cardiac electrophysiology. These successes have generally required detailed characterization of the functional outcomes of these mutations *via* classical electrophysiological methods, but similar approaches should be possible in sufficiently well-characterized neuropsychological conditions. The core requirement is a set of disease biomarkers that can be reproducibly observed in patients and a computational framework for replicating changes in those biomarkers due to testable genetic changes in the underlying neural machinery. Both of these characteristics have been developed for many congenital diseases of cardiac electrophysiology. This has been achieved in part by specifically seeking systematic understanding of diseases with clear distinguishing phenotypes, and in part through incrementally extending fundamental knowledge of cardiac bioelectricity to allow well-defined hypothesis generation and testing (126). While, as mentioned above, mental illnesses pose a greater set of challenges, there is no clear reason for these principles not to be similarly applied to the brain.

Independently of genetic diseases, current approaches to cardiac drug screening provide another example of the mature role that computation and modelling play in the development of cardiac pharmacology. At the simplest level, computational approaches have been adopted to define optimal drug characteristics for specific cardiac conditions—for example, to reduce the unintended impacts of atrial fibrillation treatments (127). Most recently and considerably, a U.S. Food and Drug Administration-initiated consortium named the Comprehensive In Vitro Proarrhythmia Assay initiative (CiPA; https://cipaproject.org/) has been created to develop a multidisciplinary platform for better discriminating compounds with likely cardiac toxicity from those that are inert or beneficial (128, 129). This is a critical societal development because cardiac toxicity is the single largest cause of advanced stage drug failure across all disease indications. As a testament to the importance of modelling in cardiac pharmacology, CiPA has been chosen as an aim to replace small mammal experimental testing with model-based computational translation of *in vitro* electrophysiology to predict drug outcomes in humans.

Finally, with respect to multiscale aspects, computational modelling of cardiac electrical pathology has advanced to the stage that patient-specific and heart-scale models are now being used to guide clinical intervention. The use of these models to predict the sites of ablation for infarct-associated arrhythmia has recently been approved for a full prospective clinical investigation by Johns Hopkins University School of Medicine. This substantial development has followed a series of compelling retrospective studies, and most recently a mixed retrospective/prospective approach (130). *In silico* studies of cellular and subcellular disease phenotypes can also be conducted on brain and heart cell models in parallel, as done in Mäki-Marttunen et al. (87).

## Experimental Validation of Biophysical Models

Taken together, biophysically detailed modelling is in many ways a promising method for creating hypotheses on the mechanisms of mental disorders with a genetic component. The obtained hypotheses need to be tested experimentally, but these experiments are difficult partly for the same reasons that make the modelling of disease phenotypes difficult: the gaps between the scales of phenomena (genes to behavior) and the translation of findings from laboratory animals to humans. One method shows a particular potential in this regard: induced pluripotent stem cell (iPSC)-based cellular models (131). The development of iPSCs provides a rare opportunity for studying neuronal excitability and identifying clinically useful biomarkers in schizophrenia based on electrophysiology (132, 133). Although human iPSC-based approaches do not provide the full complexity of the central nervous system, the cellular phenotypes are likely to lie closer to the genetic and molecular disease mechanisms than phenotypes observed at the tissue or organism level (134). Therefore, cellular and small network phenotypes, achievable in iPSC-derived neuronal two- and three-dimensional cell cultures, may offer a more direct readout of the pathophysiological processes as an intermediate step toward understanding pathophysiology at the brain level.

As the cells in the iPSC approach are derived from patients, they include the complete genetic background (and possibly epigenetic modifications) of an individual in addition to the risk alleles. On the one hand, this may make the analysis of the results more difficult, but on the other hand, it can lead to discovery of new genetic modifiers (135). Moreover, iPSC-derived neurons from patients can be transplanted into developing or mature rodent brains to provide a (non-human) brain environment into which the human neurons can structurally and functionally integrate. This procedure is costly at the moment but shows promise in preclinical research on psychotic disorders (135–139).

Finally, since mental disorders appear to be exclusively human disorders, the hypotheses concerning the disease mechanisms should also be tested directly in human patients. Invasive recordings are very seldom an option, but data from non-invasive recordings, such as EEG, magnetoencephalography (MEG), and magnetic resonance imaging (MRI), could be used for testing certain model predictions. New, automated approaches may help in model validation against clinical observations—also against EEG data (140). To obtain a better translation of simulated neuronal dynamics to clinical measurements, new computational approaches allow forward modelling of EEG and MEG data based on the description of the transmembrane currents across the neuron morphology (141). As for MRI, the link between neuronal activity and measurable MRI signals involves more unknowns and is thus more speculative, but forward models exist for this approach too (142). The use of these models is constrained by the sparse temporal resolution that is typical to MRI signals (143). On the other hand, the number of studies showing differences in structural and functional MRI data between healthy controls and people with mental disorder is steadily increasing [see, e.g., a special issue on the topic (144), and newer discoveries (145–147)]. This type of data could potentially be used to validate mechanistic multiscale models that predict differences in brain-area interactions or even development of anatomical connections between healthy controls and patients.

Having a forward model from single-cell activity (or even subcellular dynamics) to brain measurables is important, because many of the endophenotypes of mental disorders (especially those of schizophrenia) are in fact electrophysiological measures of brain activity under certain stimulus protocols (148). As an example, the mismatch negativity (MMN) is the brain’s response to an outlier among a series of stimuli, such as a sound with a slightly different pitch or duration among other identical sounds. MMN is weakened in schizophrenia patients, and it is “automatic” in the sense that it is evoked in protocols that do not require cognitive effort (149). MMN has also recently been acknowledged as a key biomarker for better understanding the pathophysiology of schizophrenia (150).

**Figure 2:**
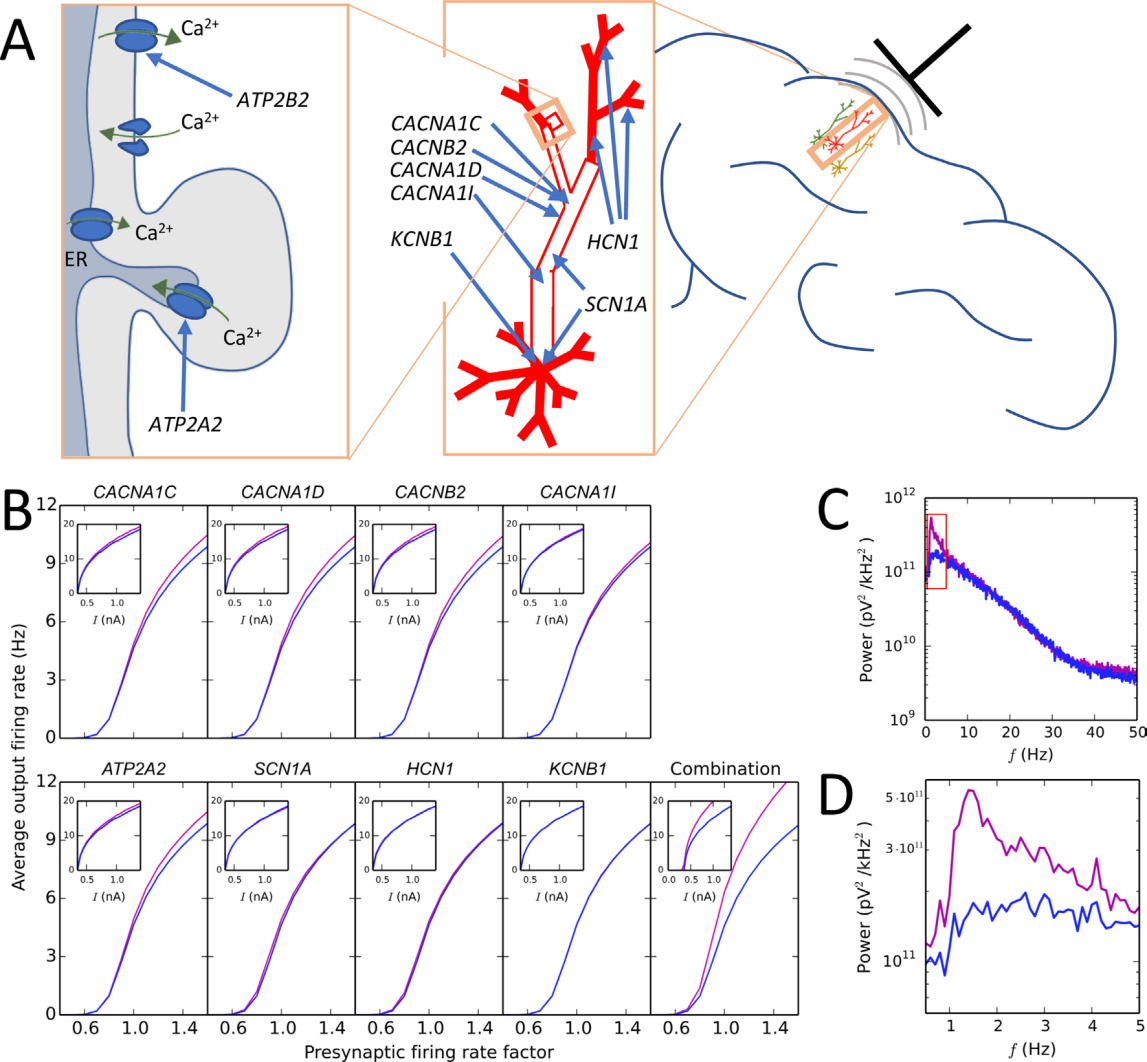
Illustration of a modelling approach predicting that a combination of small-effect variants in different schizophrenia-associated ion channel- and Ca^2+^ transporter-encoding genes causes large effects on the delta band in the EEG power spectrum. **(A)** Schematic illustration of the subcellular domain (left) of a layer V pyramidal cell (middle) and the localization of the neuron with respect to a recording electrode (right). Genes *ATP2A2* and *ATP2B2* contribute to the Ca^2+^ dynamics of the neuron: *ATP2A2* encodes a subunit of the sarco/endoplasmic reticulum Ca^2+^-ATPase (SERCA) that pumps cytosolic Ca^2+^ into the endoplasmic reticulum (ER), and *ATP2B2* encodes a subunit of the plasma membrane Ca^2+^ ATPase (PMCA) that expels cytosolic Ca^2+^ to the extracellular medium. Genes *CACNA1C*, *CACNA1D*, and *CACNB2* encode subunits of high-voltage activated Ca^2+^ channels, and *CACNA1I* encodes a low-voltage activated Ca^2+^ channel: these channels are densely expressed in the “hot zone” of Ca^2+^ channels at the apical dendrite, but they are present in the soma as well (68). *SCN1A* encodes a subunit of the fast voltage-gated Na^+^ channel that is densely expressed in the soma and mildly expressed in the apical dendrite (68). Finally, *KCNB1* encodes a subunit of a voltage-gated K^+^ channel that is only present in the soma, and *HCN1* encodes a subunit of a non-specific cationic channel whose density grows toward the end of the apical dendrite (68). **(B)** Effects of model variants of schizophrenia-associated ion channel- and Ca^2+^ transporter-encoding genes on firing rates of layer V pyramidal neurons as part of a network (main curves) and in isolation (insets). In the network experiments, interconnected networks of 150 neurons were simulated for 11 s so that the rate of presynaptic inputs (AMPA, NMDA, and GABA receptor-mediated currents) was controlled by factor (shown on *x*-axis), which influenced the average firing rate of the neurons in the network (shown on *y*-axis). In the single-cell experiments, a prolonged somatic square-pulse current (amplitude shown on *x*-axis) was given at the soma, affecting the firing frequency (shown on *y*-axis). The blue curves show the control network/neuron, while the purple curves show the network/neuron with the model variant. The fifth subpanel in the bottom row shows the effects of combination of the eight variants. For network simulations, reduced-morphology representations (48) of the layer V pyramidal cells were used to avoid excessive simulation load. Adapted from Mäki-Marttunen et al. (93). **(C)** Power spectrum of the EEG signal predicted from the activity of the network corresponding to presynaptic rate factor 1.0 in control and variant-combination conditions of panel **(B)**. To obtain this signal, transmembrane currents were registered at each compartment of each cell, and these currents were used to calculate the dipole moment time series of the cell population. From the dipole moments, the EEG signal was estimated using a theoretical model of Næss et al. (153), implemented in the LFPy Python package (141). Consistent with the predictions of panel **(B)**, the EEG power is increased for the combination of model variants compared with the control network, especially in the delta (0.5–5 Hz) range. Adapted from Mäki-Marttunen et al. (93). **(D)** Zoomed-in view on the red rectangle of panel **(B)**.

Another prominent EEG-based biomarker of schizophrenia is altered delta power, which is one of the more robust schizophrenia-associated brain-oscillation phenotypes (151). To this end, computational modellers of neuronal networks have a long history of reproducing neural oscillations of different frequencies and analyzing the cellular and network mechanisms underlying them (152). **Figure 2** shows findings from our recent study where the forward EEG modelling approach was used to show that alterations in schizophrenia-associated voltage-gated ion channel and Ca^2+^ transporter-encoding genes can cause increased delta power (93). Discovering the mechanisms through which genetic risk leads to these widely examined quantitative biomarkers is an important milestone on the path to understanding the complex, largely qualitative symptoms and pathogenesis of mental disorders. In addition to EEG protocols based on resting state or sensory stimuli, magnetic and electrical brain stimulation could also be used for testing hypotheses on altered brain activity in mental disorders [these approaches are reviewed in Ref. (102)].

## Relevance for Biomarkers and Drug Development

In addition to shedding light on the disease mechanisms of mental disorders, biophysically detailed neuron modelling is a suitable tool to assist drug development for diseases where ion-channel functions are impaired (cf.^154^, ^155^). However, until a clearer picture of the pathology of a mental disorder is formed, the use of biophysical modelling as a means of treatment design may remain a long-term ambition. Especially in the case of schizophrenia, there are currently many more open questions concerning all levels of brain function than answers to these questions: How do the schizophrenia-associated gene variants (27) affect neuronal excitability in different brain areas? Are the observed changes in connectivity a response to this altered excitability, or do they represent an independent disease phenotype? How can we relate changes in neuron excitability and neuronal connectivity to negative and positive symptoms of the disease? What is the cell-type specific contribution to the pathophysiology? If the impaired ion-channel function could be restored by drugs, should it be done before the onset of the disease or can it be done afterwards? From the modelling perspective, the involvement of the immune system in schizophrenia represents another great unknown, as there are few modelling efforts integrating the neuronal functions with altered immune system functions. Nevertheless, in combination with animal models of schizophrenia, computational models could be employed to test specific hypotheses on drug effects on cellular and network-level functions, and the data obtained could be used to translate them into hypotheses on human brain functions.

We believe that in the near future, biophysically detailed modelling can become a fruitful tool in systematic analysis of the endophenotypes and biomarkers of the mental disorders. Endophenotypes have been suggested as a gate toward understanding the pathology of diseases where both the genetic origin and disease symptoms are complex (156). This is a case in point for schizophrenia. There are many widely adopted schizophrenia-associated endophenotypes and biomarkers that can be quantified using behavioral tests or electrophysiology. Due to the possibility of reference to animal models, these endophenotypes and biomarkers are better characterized than the symptoms of schizophrenia, which are almost exclusively human symptoms. The genetic foundation of these endophenotypes and biomarkers is still largely unknown. However, unlike the symptoms of the mental disorders, the physiology and neuronal origin of these phenotypes are relatively well mapped. Biophysically detailed modelling could become an invaluable tool for deeper characterization of the effects of disease-associated variants on the disease endophenotypes and biomarkers, which would shed light on the pathology of the heritable mental disorder as a whole.

## Conclusions

Biophysically detailed modelling of brain functions and the contributions of disease-associated genetic variants therein is a significant challenge for this century. Eventually, this “biophysical psychiatry” approach may become a powerful approach to generate testable hypotheses for the pathophysiology of complex, heritable mental disorders such as schizophrenia. International consortia that collect and analyze patient data as well as other large-scale efforts that map mammalian brain structure and function are key sources of data for this line of research, but new innovative experimental techniques and modelling approaches are required as well in order to gain maximally informative insights into mental disorder pathology.

## Author Contributions

TM-M, AE, GE, and OA wrote the manuscript. TK, TE, AD, SD, LW, M-LL, MR, DS, SB, ME-B, FB, GH, EH, SN, TN, TM, CM, MF, and AD provided analytical support.

## Funding

Funding for this work was received through NIH grant 5 R01 EB000790-10, the European Union Horizon 2020 Research and Innovation Programme under Grant Agreement No. 785907 [Human Brain Project (HBP) SGA2]; ERA-NET NEURON project “SYNSCHIZ” (Research Council of Norway, grant number 283798; Bundesministerium für Bildung und Forschung, grant number 01EW1810; Swiss National Science Foundation, grant number 32NE30 179602; Academy of Finland, grant number 318879; NWO & Hersenstichting, grant number 013-17-003 4538; UEFISCDI, grant number 6/2018); Research Council of Norway (216699, 223273, 226971, 248778, 249711, 248828, and 250128); South East Norway Health Authority (2016-082,2017-112); KG Jebsen Stiftelsen, and a research grant from Mrs. Throne-Holst.

## Conflicts of Interest Statement

The authors declare that the research was conducted in the absence of any commercial or financial relationships that could be construed as a potential conflict of interest.
